# Hypoxia-inducible transcription factor-1α promotes hypoxia-induced A549 apoptosis via a mechanism that involves the glycolysis pathway

**DOI:** 10.1186/1471-2407-6-26

**Published:** 2006-01-27

**Authors:** FengMing Luo, XiaoJing Liu, NaiHong Yan, ShuangQing Li, GuiQun Cao, QingYing Cheng, QingJie Xia, HongJing Wang

**Affiliations:** 1 West China Hospital, Sichuan University, Chengdu, China; 2 West China Second University Hospital, Sichuan University, Chengdu, China

## Abstract

**Background:**

Hypoxia-inducible transcription factor-1α (HIF-1α), which plays an important role in controlling the hypoxia-induced glycolysis pathway, is a "master" gene in the tissue hypoxia response during tumor development. However, its role in the apoptosis of non-small cell lung cancer remains unknown. Here, we have studied the effects of HIF-1α on apoptosis by modulating HIF-1α gene expression in A549 cells through both siRNA knock-down and over-expression.

**Methods:**

A549 cells were transfected with a HIF-1α siRNA plasmid or a HIF-1α expression vector. Transfected cells were exposed to a normoxic or hypoxic environment in the presence or absence of 25 mM HEPES and 2-deoxyglucose (2-DG) (5 mM). The expression of three key genes of the glycolysis pathway, glucose transporter type 1(GLUT1), phosphoglycerate kinase 1(PGK1), and hexokinase 1(HK1), were measured using real-time RT-PCR. Glycolysis was monitored by measuring changes of pH and lactate concentration in the culture medium. Apoptosis was detected by TUNEL assay and flow cytometry.

**Results:**

Knocking down expression of HIF-1α inhibited the glycolysis pathway, increased the pH of the culture medium, and protected the cells from hypoxia-induced apoptosis. In contrast, over-expression of HIF-1α accelerated glycolysis in A549 cells, decreased the pH of the culture medium, and enhanced hypoxia-induced apoptosis. These effects of HIF-1α on glycolysis, pH of the medium, and apoptosis were reversed by treatment with the glycolytic inhibitor, 2-DG. Apoptosis induced by HIF-1α over-expression was partially inhibited by increasing the buffering capacity of the culture medium by adding HEPES.

**Conclusion:**

During hypoxia in A549 cells, HIF-1α promotes activity of the glycolysis pathway and decreases the pH of the culture medium, resulting in increased cellular apoptosis.

## Background

Hypoxic regions are a common feature within rapidly growing malignant tumors such as non-small cell lung cancer (NSCLC). Tissue hypoxia caused by inadequate blood supply occurs very early during tumor development, usually when the tumor diameter is just a few millimeters [1]. Therefore, over-expression of hypoxia-inducible transcription factor-1 (HIF-1), a "master" gene in the hypoxic response, is a frequent occurrence in many tumor cells, including NSCLC [2]. However, the role of HIF-1 in NSCLC remains controversial. The HIF-1 complex is a heterodimer consisting of a constitutively expressed HIF-1β subunit and a HIF-1α subunit, which is highly transcriptionally regulated, as well as being susceptible to oxygen-dependent degradation of the protein [3]. Giatromanolaki et al [4] reported that HIF-1α over-expression is common in NSCLC, and that this is related to the up-regulation of various angiogenic factors and associated with poor prognosis. They also suggested that HIF pathway might be an important therapeutic target for NSCLC. However, these results are at odds with those of Volm et al [5], who demonstrated that NSCLC patients with HIF-1α-positive carcinomas had significantly longer median survival times than patients with HIF-1α-negative carcinomas. Yet another group [6] confirmed that down-regulation of HIF-1 abrogates hypoxia-induced cell growth arrest. More recently, Yu et al [7] reported that HIF-1α exerted an anti-apoptotic role in human umbilical vascular endothelial cells (HUVECs) under anoxic stress. As apoptosis is one of the most important mechanisms mediating cancer remission, investigation into the role of HIF-1α in apoptosis of lung cancer may provide useful information for the resolution of these controversies.

As a key mediator of the hypoxic response in animal cells, HIF-1 plays an important role in controlling the glycolysis pathway when cells are exposed to low oxygen tension. During hypoxia, cells shift to a primarily glycolytic metabolic mode by up-regulating HIF-1-dependent glycolytic genes, such as glucose transporter type 1(GLUT1), phosphoglycerate kinase 1(PGK1), hexokinase 1(HK1), etc [3,8,9]. In addition, intracellular pH decreases as a result of lactate acid accumulation caused by accelerated glycolysis. Because intracellular acidosis triggers apoptosis [10], blocking increased glycolytic activity by down regulating HIF-1α may reduce apoptosis of the hypoxic cells.

We investigated whether controlling HIF-1α expression regulated apoptosis of the lung adenocarcinoma cell line, A549, during hypoxia, and whether this effect of HIF-1α was dependent upon glycolysis. The research presented in this report addresses these fundamental questions about the role of HIF-1 in the pathophysiology of NSCLC.

## Methods

### Plasmid construction

siRNA plasmid vectors were constructed as previously described [11]. Pairs of annealed DNA oligonucleotides were inserted between the *Bgl*II and *Hind*III restriction sites of the EGFP-pSUPER vector (a gift from Dr. Chunlin Chen, the third Military Medicine University, China) in order to express short hairpin small interfering RNA (siRNA) under the control of the polymerase-III H1-RNA promoter. A nineteen-nucleotide (nt) target sequence derived from human HIF-1α mRNA (accession #U22431; bp 1470–1489) was selected as previously described [11]. A set of 64-nt oligos containing this sequence are described below: HIF-1α siRNA forward oligo,5'-gatccccATCCAGAGTCACTGGAACTttcaagaga AGTTCCAGTGACTCTGGATtttttggaaa-3'; reverse oligo, 5'-agcttttccaaaaa ATCCAGAGTCACTGGAACTtctcttgaaAGTTCCAGTGACTCTGGAggg-3'. A scrambled sequence control siRNA primer set was also designed: forward oligo, 5'-gatccccGACCGCAATATGGCACTTAttcaagagaTAAGTGCCATATTGCGGTCtttttggaaa-3'; reverse oligo, 5'-agcttttccaaaaaGACCGCAATATGGCACTTAtctcttgaa TAAGTGCCATATTGCGGTCggg-3'. The target sequences of the HIF-1α siRNA and scrambled HIF-1α control siRNA were BLAST searched against the GenBank database. The HIF-1α targeting sequence matched exactly with partial sequences of the human HIF-1α gene, but not with any other genes. The scrambled control did not match any known human gene. Constructs expressing HIF-1α (wildtype) were a gift from Professor Cormac T. Taylor (University College, Dublin, Ireland).

### Cell culture

A549 cells were obtained from the American Type Culture Collection, (Manassas, VA, USA). Cells (at 1 × 10^5 ^cells/ml) were plated in tissue culture flasks and incubated in normoxia (21% O_2_,74% N2 and 5% CO_2_) at 37°C in DMEM medium (Invitrogen, Carlsbad, CA, USA) supplemented with 10% heat-inactived fetal bovine serum (Invitrogen) and penicillin-streptomycin (50 μg/ml, Invitrogen). A549 cells were then plated onto 6-well, flat-bottom tissue culture plates (Becton Dickinson and Co., NJ, USA) at a density of 1 × 10^5 ^cells/well in DMEM medium. Hypoxic conditions were achieved by culturing cells in an incubator (Jouan, Saint-Nazaire, France.) with a 1% O2, 5% CO2, and 94% N2 atmosphere. Acidosis was neutralized by addition of 25 mM HEPES (Sigma-Aldrich Corp. St. Louis, MO, USA) buffer so as to increase the buffering capacity of the culture media[10].

### Transient transfection

Transient transfections were performed using the cationic lipid, Lipofectamine 2000 (Invitrogen), according to the manufacturer's specifications. All of these experiments were performed in 6-well tissue culture plates with cells plated to reach 50–60% confluence on the day of transfection. Transfection efficiency averaged between 60–70%, as measured by green fluorescent protein expression. Cells were allowed to recover in DMEM for 20 h after transfection, and then the medium was changed and the culture was exposed to normoxia, hypoxia, or the glycolytic inhibitor 2-DG (5 mM, Sigma-Aldrich Corp [12] for 24 h before assays were performed.

### RNA extraction and real-time RT-PCR

RNA extraction and real-time PCR were performed as previously described [13-15]. Briefly, total cellular RNA was extracted using an acid guanidinium-phenol-chloroform method (TRIzol^®^; Invitrogen). RNA integrity was confirmed by electrophoresis on ethidium bromide-stained 1% agarose gels. Total cellular RNA, 1 μg, was reverse transcribed at 37°C for 70 min in 20 μl containing 2.5 U reverse transcriptase (Fermentas Inc. Unit A Hanover, MD, USA), 10 mM dithiothreitol, 1 mM each of deoxyadenosine triphosphate (dATP), deoxythymidine triphosphate (dTTP), deoxycytidine triphosphate (dCTP), and deoxyguanidine triphosphate (dGTP), and 5 μg/ml oligo-dT primer (Fermentas Inc.). Reactions were stopped by heat inactivation for 10 min at 85°C. Primers were synthesized and HPLC purified (Takara, Dalian, China). Primer sequences used for amplification were as follows: HIF-1α: upstream primer, 5'-CTG ACC CTG CAC TCA ATC AA-3'; downstream primer, 5'-CTT TGC TTC TGT GTC TTC AGC AGC A-3'; HIF1-1α probe: 5'-FAM-CAC CTG AGC CTA ATA GTC CCA G-3'; GLUT1: upstream primer, 5'-CTTTGTGGCCTTCTTTGAAGT-3'; downstream primer, 5'-CCACACAGTTGCTCCACAT-3'; GLUT1 probe: 5'-FAM-CAGGCT TCTCCAACTGGACCTC-TAMRA-3'; HK1: upstream primer, 5'-TCCGTAGTGGGAAAAAGAGAA-3'; downstream primer, 5'-GACAATG TGATCAAACAGCTC-3'; HK1 probe: 5'-FAM-CACAACAAGATCTACGC CATTCC-TAMRA-3'; PGK1: upstream primer, 5'-CCACTTGCTGT GCCA AATGGA-3'; downstream primer, 5'-GAAGGACTTTACCTTCCAG GA-3'; PGK1 probe: 5'-FAM-CCAGTGCTC ACATGGCTGAC-3'; β-actin: upstream primer, 5'-GCCAACACAGTGCTGTCT-3'; downstream primer, 5'-AGGAGC AATGATCTTGATCTT-3'; β-actin probe: 5'-FAM-ATCTCCTTCTGCATCCT GTC-3'. Real-time PCR was performed on an ABI Prism 7700 sequence detection system (PE Applied Biosystems). The reactions were cycled 40 times after initial denaturation (95°C, 2 min) using the following parameters: denaturation, 95°C for 15 s and annealing and extension, 60°C for 1 min. The threshold cycle (Ct) was recorded for each sample to reflect the mRNA abundance level. The mRNA expression indexes of the three target genes were calculated as previously described [15].

### Western-blot analysis

For Western blot analysis, normoxic or hypoxic cells were scraped from dishes and cellular protein extracts were prepared by homogenization in ice cold lysis buffer (0.5 M Tris, 250 mM NaCl, 0.1% Nonidet P-40, 0.2 M Na3VO4, 0.2 M NaF) containing a protease inhibitor cocktail (Sigma-Aldrich Chemie GmbH, Deisenhofen, Germany). Forty micrograms of protein were separated per lane by 12% sodium dodecyl sulfate-polyacrylamide gel electrophoresis (SDS-PAGE). After electrophoretic transfer of proteins to a polyvinylidene difluoride (PVDF) membrane (Millipore, Bedfordshire, United Kingdom), HIF-1α specific bands were detected by reaction with a rabbit antibody against HIF-1α (Santa Cruz Biotechnology, Inc., Santa Cruz, CA, USA), followed by horseradish peroxidase-conjugated anti-rabbit secondary antibody. Bands were visualized by enhanced chemiluminescence (ECL, Amersham, Freiburg, Germany). Immunoblots were stripped using mild antibody stripping solution and re-probed with an anti-actin antibody (Santa Cruz Biotechnology, Inc.). For quantification purposes, densitometric measurements were performed using the Quantity One image analysis software for Windows (Bio-Rad, Alfred Nobel Drive Hercules, CA, USA). All HIF-1α values were normalized to actin levels.

### pH measurement

For pH measurement, cells were cultured using medium with or without HEPES. Immediately after hypoxic challenge, the pH of the medium was measured withan electronic pH meter (pH meter 420; Corning, Corning, NY, USA). Measurements were made within 1 min after the termination of hypoxic treatment.

### Lactate concentration measurements

A549 cell culture media were collected for lactate release measurements. Lactate concentrations were determined using a commercial kit (Nanjing Jiancheng Bioengineering Institute, Nanjing, China). The intra-assay coefficient of variation was 1.1%.

### Detection of apoptosis using flow cytometry

Adherent cells were released from the plate with 5 mM EDTA and collected. Floating cells were also recovered and included with the formerly adherent cells for the apoptosis assay. Following two washes by PBS, cells were permeabilized in 100 μg/ml digitonin solution at 4°C for 20 min. Cells were then washed and stained with 2 μg/ml of Apo2.7 monoclonal antibody for 15 min. The Apo2.7 antibody (Coulter Corp, Cedex, France) reacts with a 38-kDa mitochondrial membrane protein (7A6 antigen), which is expressed at an early stage of apoptosis, thus marking the process of apoptosis itself rather than simply the end point of dead cells [16]. Cells were finally washed with 1.0 ml of PBS with 2.5% fetal calf serum and stored in the dark on ice until assayed.

### Terminal deoxynucleotidyl transferase-mediated dUTP nick end labeling (TUNEL) assay

The TUNEL technique was performed to detect and quantitate apoptotic cell death using the In situ Cell Death Detection Kit (Roche Diagnostics, Indianapolis, IN, USA), as reported previously [7]. Briefly, cover slides were fixed with 4% paraformaldehyde for 1 h and permeabilized in 0.1% Triton-100, 0.1% sodium citrate at 4°C for 2 min. The slides were incubated with TUNEL reaction mixture for 1 h at 37°C. After washing, the slides were incubated with alkaline phosphatase-conjugated anti-fluorescein antibody for 30 min at 37°C. Slides were lightly counterstained with hematoxylin.

### Statistical analysis

The experimental data were expressed as mean ± SD. Group means were compared by One-way ANOVA using the statistical software program SPSS 10.0 for windows (Chicago, IL, USA). *P *values < 0.05 were considered significant in all cases.

## Results

### Down-regulation of HIF-1α gene expression in A549 cells with siRNA

**Figure 1:**
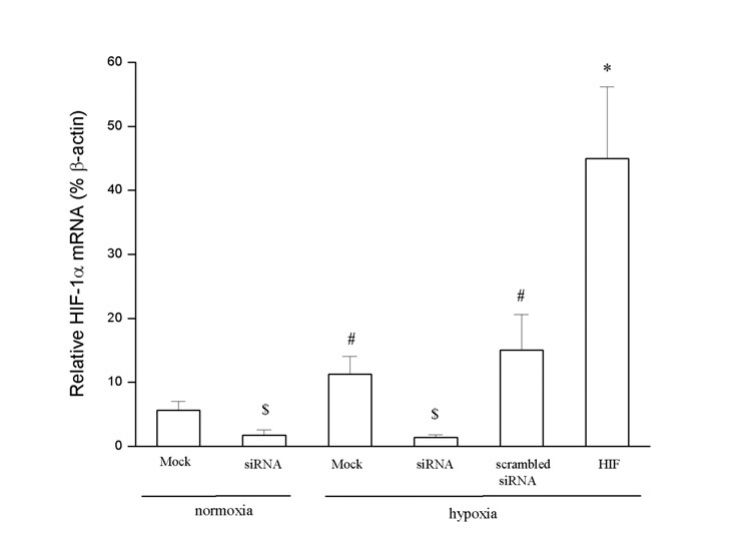
**Inhibition HIF-1α mRNA expression in A549 by siRNA**. A549 were transfected with the siRNA-expressing EGFP-pSUPER vector or a HIF-1 expression plasmid. Cells were allowed to recover in regular culture medium for 20 h after transfection, then exposed to a normoxic or hypoxic environment for an additional 24 h. Experiments were performed five times with the similar results (n = 5 in each group). $, P < 0.05 compared to control and HIF-1α siRNA; #, P < 0.05 compared to normoxic or hypoxic HIF-1α siRNA; *, P < 0.05 compared to hypoxic mock-transfected and scrambled HIF-1α control siRNA transfection. *siRNA: HIF-1α plasmid transfected, Mock: mock transfected, HIF: HIF-1α expression plasmid transfected*.

**Figure 2:**
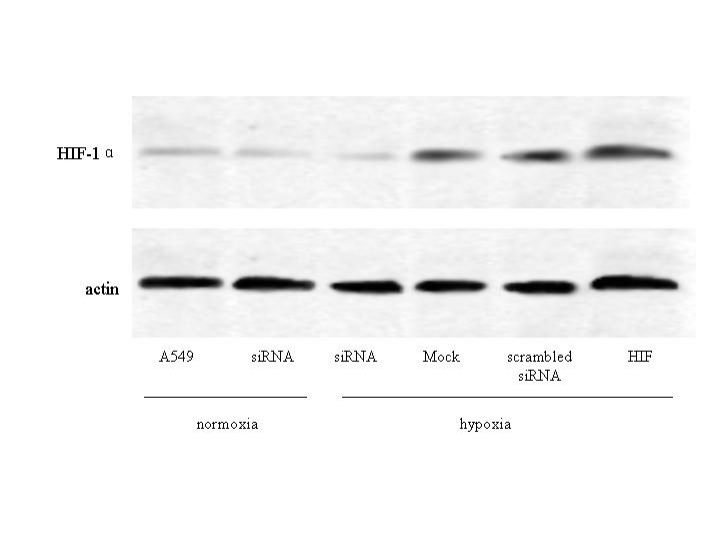
**Suppression and over-expression of HIF-1α: western analysis**. A549 cells were transfected with HIF-1α siRNA vector or a HIF-1 expression plasmid. Cells were allowed to recover in regular culture medium for 20 h after transfection, then exposed to normoxia or hypoxia for 24 h, at which point cellular protein extracts were prepared (n = 5 in each group). Proteins were separated by SDS-PAGE and transferred to membrane for western blot analysis. *siRNA: HIF-1α plasmid transfected, Mock: mock transfected, HIF: HIF-1α expression plasmid transfected*.

**Figure 3:**
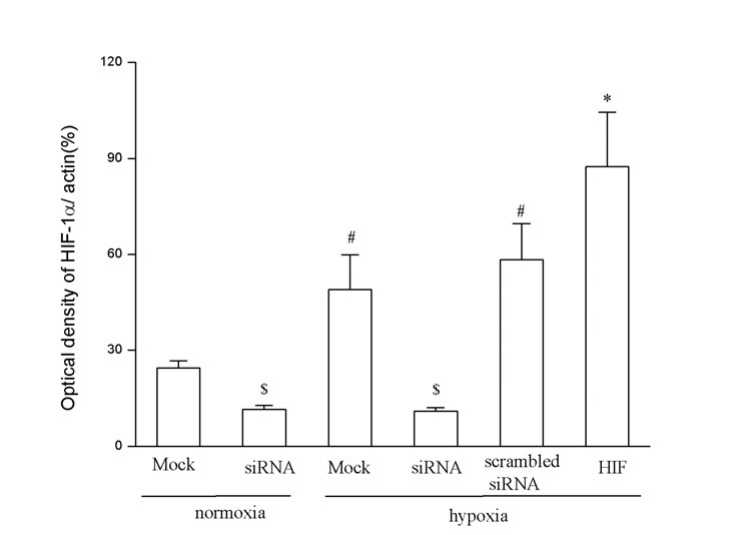
**Suppression and over-expression of HIF-1α: graphical format**. Densitometric analysis was performed on the western blot shown in figure 3 and displayed here in graphical format. $, P < 0.05 compared to control and HIF-1α siRNA; #, P < 0.05 compared to normoxic and hypoxic HIF-1α siRNA; *, P < 0.05 compared to hypoxic mock transfected and scrambled HIF-1α control siRNA transfection. *siRNA: HIF-1α plasmid transfected, Mock: mock transfected, HIF: HIF-1α expression plasmid transfected*.

As shown in Figure 1, in A549 cells transfected with the siRNA EGFP-pSUPER vector system, the hypoxia-induced expression of HIF-1α mRNA was significantly suppressed. Accordingly, protein levels were likewise decreased in cells receiving this siRNA (Figure 2 and 3). Conversely, transfection of A549 with a HIF-1α expression vector led to up-regulation HIF-1α at both the mRNA and protein level during hypoxia (Figure 1, 2 and 3).

### Suppressed HIF-1α expression led to the decreased expression of key glycolysis genes

**Figure 4:**
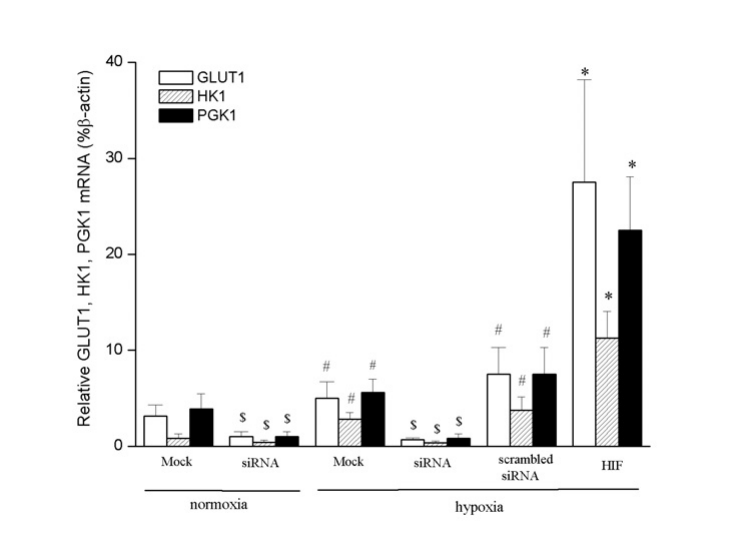
**siRNA-mediated knock-down of HIF-1α expression leads to the suppression of three key glycolysis genes**. Expression of three key genes in the glycosis pathway, GLUT1, HK1, and PGK1 was monitored by real-time RT-PCR. Cells were transfected with HIF-1α siRNA EGFP-pSUPER vector, scrambled HIF-1α control siRNA, or an expression plasmid encoding HIF-1α. After 20 h, transfected cells were exposed to hypoxia for 24 h (n = 5 in each group). $, P < 0.05 compared to normoxic control; #, P < 0.05 compared to normoxic and hypoxic HIF-1α siRNA; *, P < 0.05 compared to hypoxic mock-transfected and scrambled HIF-1α control siRNA transfection. *siRNA: HIF-1α siRNA vector transfected, Mock: mock transfected, HIF: HIF-1α expression plasmid transfected*.

To investigate the role of HIF-1α in the regulation of glycolysis during hypoxia, message levels three key genes in this pathway were quantitated by real-time RT-PCR. Hypoxia increased the abundance of HIF-1α mRNA, as well as three key glycolysis genes, GLUT1, HK1, and PGK1. When HIF-1α expression was knocked-down with specific siRNA, expression of all three glycolytic genes was inhibited. In contrast, when the reverse condition, over-expression of HIF-1α, was applied, expression of these three genes increased in A549 cells (Figure 4).

### HIF-1α is involved in the regulation of glycolysis during hypoxia

**Figure 5:**
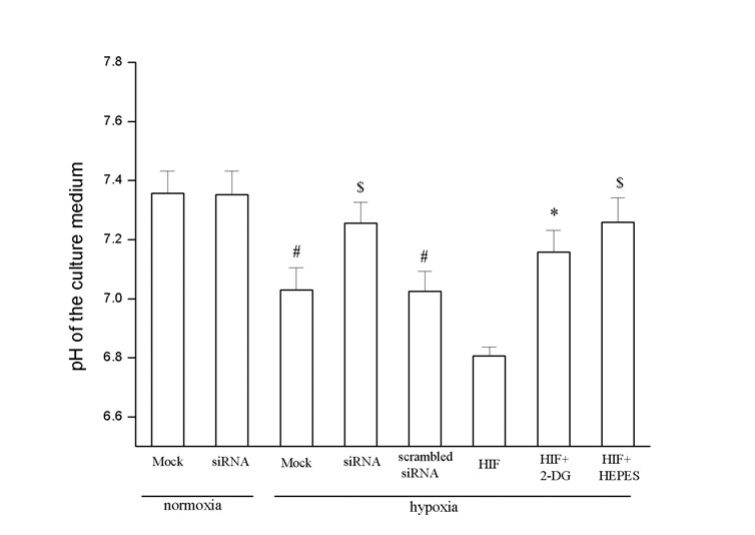
**Quantitation of the cell culture medium pH**. The media from cell cultures were collected after transfection of the cells with HIF-1α siRNA vector or a HIF-1α expression plasmid and exposure to hypoxic conditions for 24 h. The pH of the cell culture medium measured immediately after samples collected (n = 5 in each group). $, P < 0.05 compared to normoxic mock and HIF-1α siRNA transfected; *, P < 0.05 compared to hypoxic HIF-1α siRNA and HIF-1α expression plasmid with HEPES; #, P < 0.05 compared to hypoxic HIF-1αexpression plasmid with or without 2-DG. *siRNA: HIF-1α siRNA EGFP-pSUPER vector transfected, Mock: mock-transfected, HIF: HIF-1α expression plasmid transfected, HIF+2-DG: Cells were transfected with HIF-1α expression plasmid and cultured with 2-DG, HIF+HEPES: Cells were transfected with HIF-1α expression plasmid and cultured with medium containing HEPES*.

**Figure 6:**
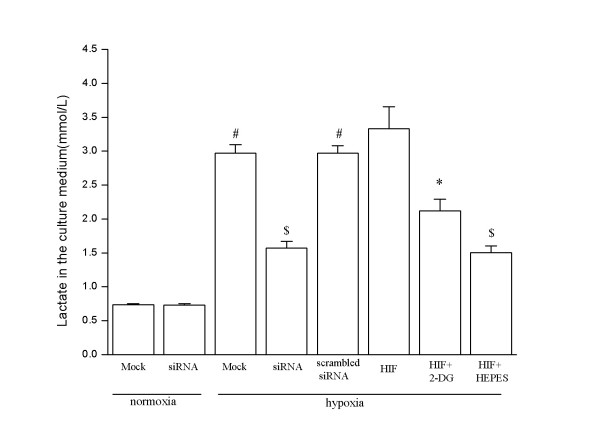
**Measurement of lactate levels in the cell culture medium**. The media from cell cultures were collected after transfection of the cells with HIF-1α siRNA vector or a HIF-1α expression plasmid and exposure to hypoxic conditions for 24 h. Lactate concentration was measured using a commercial kit (n = 5 in each group). $, P < 0.05 compared to normoxic mock transfected and HIF-1α siRNA; *, P < 0.05 compared to hypoxic HIF-1α siRNA and HIF-1α expression vector with HEPES; #, P < 0.05 compared to hypoxic HIF-1α expression vector with or without 2-DG. *siRNA: A549 cells transfected HIF-1α siRNA vector, Mock: mock-transfected, HIF: A549 cells transfected with HIF-1α expression plasmid, HIF+2-DG: Cells transfected with HIF-1α expression plasmid and cultured with 2-DG, HIF+HEPES: Cells transfected with HIF-1α expression plasmid and cultured with medium containing HEPES*.

To further investigate the role of HIF-1α in glycolysis of A549 cells during hypoxia, we monitored pH and the final product of glycolysis, lactate, in the cell culture medium. A hypoxic environment decreased the pH of the culture medium, while increasing the concentration of lactate. Consistent with a role for HIF-1α in this process, transfection with siRNA specific for this gene significantly restored normal pH and decreased lactate levels compared to a scrambled siRNA control. However, under normoxia, over-expression of HIF-1α in A549 cells led to decreased pH and increased lactate concentration in the culture medium (Figures 5 and 6). Under hypoxic conditions, addition of 2-DG prevented both acidification of the medium and the accumulation of lactate induced by over-expression of HIF-1α. The pH of medium supplemented with 25 mM HEPES was higher than un-supplemented medium after 24 h exposure to hypoxia.

### HIF-1α initiates hypoxia-induced apoptosis in A549 cells

**Figure 7:**
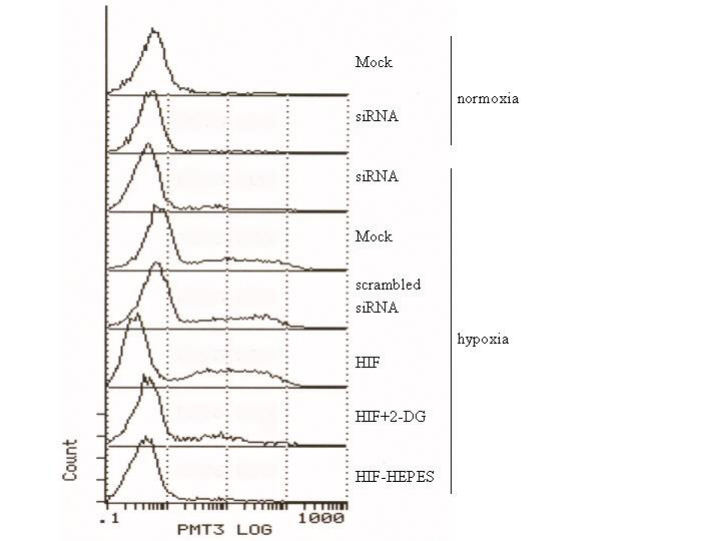
**Detection of apoptosis by flow cytometry: histogram analysis**. A549 cells were transfected with HIF-1α siRNA vector or HIF-1 expression plasmid. Cells were allowed to recover in regular culture medium for 20 h after transfection, then exposed to normoxic or hypoxic environment for 24 h with or without additional HEPES (25 mM) or the glycolytic inhibitor, 2-DG (5 mM) (n = 5 in each group). Cells were collected and stained with Apo2.7 monoclonal antibody (7A6). Shown are representative results from one of five independent experiments. *siRNA: A549 cells transfected HIF-1α siRNA vector, Mock: mock-transfected, HIF: A549 cells transfected with HIF-1α expression plasmid, HIF+2-DG: Cells transfected with HIF-1α expression plasmid and cultured with 2-DG, HIF+HEPES: Cells transfected with HIF-1α expression plasmid and cultured with medium containing HEPES*.

**Figure 8:**
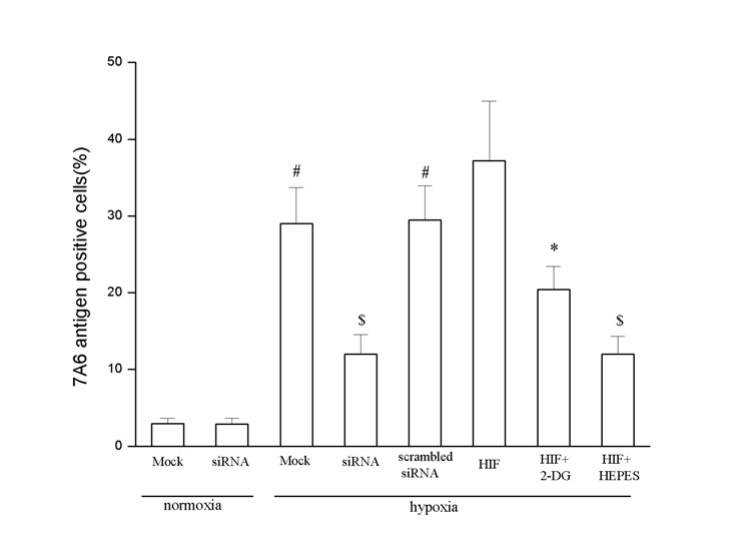
**Detection of apoptosis by flow cytometry: graphical format**. Cells were treated and stained as described for Fig. 7. Shown is a graphical representation of this data with statistical analysis from a representative of 5 independent experiments. $, P < 0.05 compared to normoxic mock transfected and HIF-1α siRNA; *, P < 0.05 compared to hypoxic HIF-1α siRNA and HIF-1α expression vector with HEPES; #, P < 0.05 compared to hypoxic HIF-1α expression vector with or without 2-DG. *siRNA: A549 cells transfected HIF-1α siRNA vector, Mock: mock-transfected, HIF: A549 cells transfected with HIF-1α expression plasmid, HIF+2-DG: Cells transfected with HIF-1α expression plasmid and cultured with 2-DG, HIF+HEPES: Cells transfected with HIF-1α expression plasmid and cultured with medium containing HEPES*.

**Figure 9:**
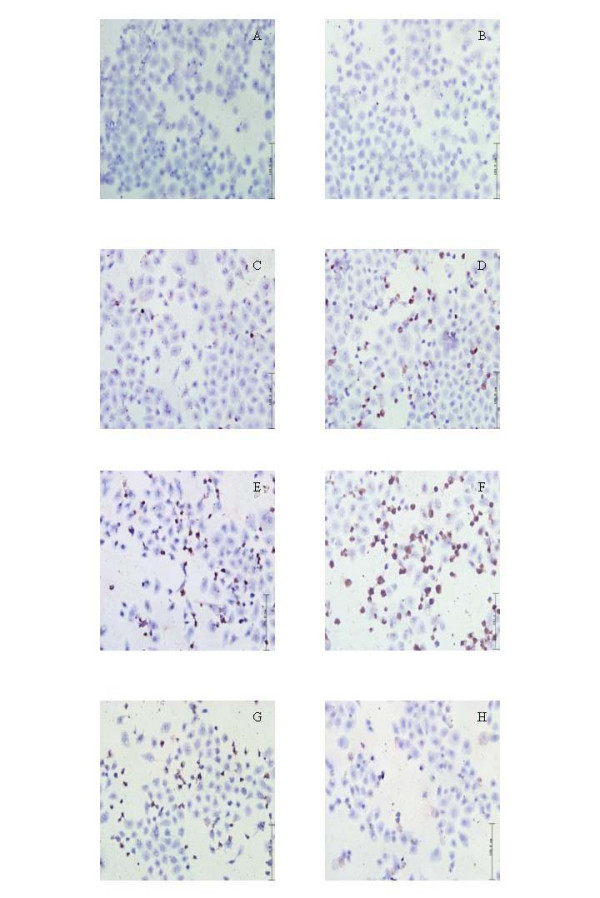
**Detection of apoptosis by TUNEL: Staining**. A549 cells were cultured on cover slides and transfected with different plasmids. Cells were allowed to recover in regular culture medium for 20 h after transfection, then exposed to normoxic or hypoxic atmosphere for 24 h with or without HEPES (25 mM) or the glycolytic inhibitor, 2-DG (5 mM) (n = 5 in each group). Only occasional normoxic cells were positive for TUNEL staining, (A). Hypoxia increased the amount of apoptosis of A549 cells, (D). Over-expression of HIF-1α further increased this pro-apoptotic effect of hypoxia, (F). However, siRNA suppression of HIF-1α expression rescued A549 cells from hypoxia-induced apoptosis, (C); but scrambled siRNA did not have this effect, (E). The pro-apoptotic effect of HIF-1α was also inhibited by the glycolytic inhibitor, 2-DG, (G). Increasing the buffering capacity of the culture medium with HEPES also inhibited the pro-apoptotic effect of HIF-1α, (H). Shown are representative photomicrographs from five independent experiments. Figure 9 was statistics of the five independent experiments. *siRNA: A549 cells transfected HIF-1α siRNA vector, Mock: mock-transfected, HIF: A549 cells transfected with HIF-1α expression plasmid, HIF+2-DG: Cells transfected with HIF-1α expression plasmid and cultured with 2-DG, HIF+HEPES: Cells transfected with HIF-1α expression plasmid and cultured with medium containing HEPES. Scale bar: 100 μm*.

**Figure 10:**
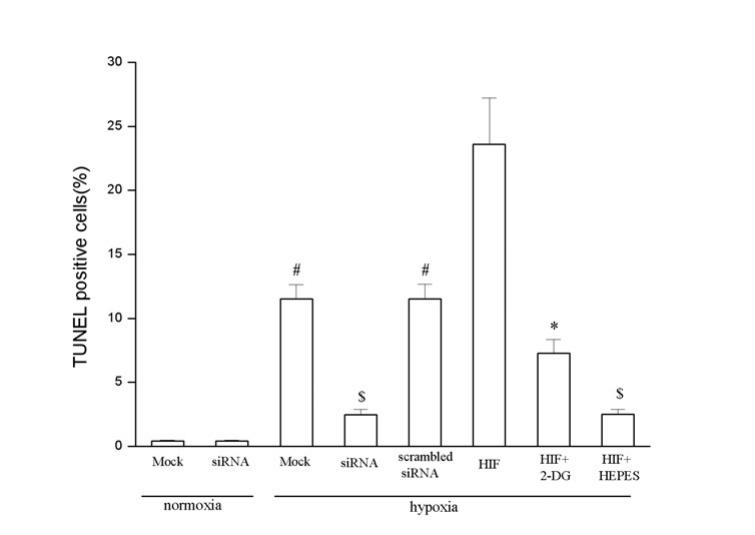
**Detection of apoptosis by TUNEL: graphical format**. TUNEL staining was performed on cells as described above. Shown is a graphical representation of the staining shown in Fig. 9. $, P < 0.05 compared to normoxic mock transfected and HIF-1α siRNA; *, P < 0.05 compared to hypoxic HIF-1α siRNA and HIF-1α expression vector with HEPES; #, P < 0.05 compared to hypoxic HIF-1α expression vector with or without 2-DG. *siRNA: A549 cells transfected HIF-1α siRNA vector, Mock: mock-transfected, HIF: A549 cells transfected with HIF-1α expression plasmid, HIF+2-DG: Cells transfected with HIF-1α expression plasmid and cultured with 2-DG, HIF+HEPES: Cells transfected with HIF-1α expression plasmid and cultured with medium containing HEPES*.

Hypoxic conditions induced apoptosis of A549 cells, as detected by flow-cytometry and TUNEL assays. Over-expression of HIF-1α further exacerbates hypoxia-induced apoptosis. In contrast, siRNA-mediated suppression of HIF-1α expression rescued A549 cells from apoptotic death induced by hypoxia (Figure 7, 8, 9, 10). The pro-apoptotic activity of HIF-1α was effectively countered by the glycolytic inhibitor, 2-DG (Figure 7, 8, 9, 10), as well as by increasing the buffering capacity of the culture medium (Figure 7, 8, 9, 10).

## Discussion

HIF-1α is a "master" gene controlling the hypoxic response in mammalian cells and thus plays an important role in tumor growth. In this study, we demonstrated that modulating the expression level of HIF-1α was able to increase or decrease apoptosis of a tumor cell line, A549, under hypoxic stress. Furthermore, this effect of HIF-1α was dependent upon the glycolysis pathway and pH changes of the culture medium, both of which appear to be regulated by HIF-1α during hypoxia.

During hypoxia, transportation of glucose into the cell and glycolytic enzyme activity are enhanced. Consistent with previous studies, we also found that hypoxia up-regulated expression of two key genes in the glycolysis pathway, HK1 and PGK1, as well as a glucose transportation gene, GLUT1 [3,8,9]. These genes were responsive to the level of HIF-1α, as down-regulation of HIF-1α with siRNA decreased their expression, while up-regulation of HIF-1α with an expression vector similarly increased expression of GLUT1, HK1, and PGK1. Taken together, our results indicate that the expression of key genes controlling glucose transportation and glycolysis are under the control of HIF-1α during hypoxia. Furthermore, over-expression HIF-1α in hypoxic A549 cells resulted in increased accumulation of lactate and decreased pH in the culture medium. When HIF-1α expression was inhibited with siRNA during hypoxia in the converse experiment, lactate levels instead decreased, accompanied by a restoration of normal pH in the culture medium. As lactate is the final product of glycolysis, increased glycolysis is expected to lead to acidosis of cell culture medium during hypoxia. Therefore, lactate accumulation and acidic pH may be an indicator of increased glycolytic activity. Thus, based on the result showing that HIF-1α regulates the expression of genes involved in both glucose transportation and glycolysis described above, we conclude that HIF-1α regulates the glycolysis pathway of A549 cells during hypoxia.

It is important to note that in another model, hypoxic rat cardiomyocytes, glucose-uptake and metabolism was found to be protective against hypoxia-induced apoptosis [17]. In that study, glycolysis was shown to account for the protective effects, but other metabolic substrates provided no such protection from apoptosis [17]. In contrast to this, the study presented here found that HIF-1α contributes to up-regulation of the glycolysis pathway of A549 cells during hypoxia. Furthermore, this increased metabolic activity leads to hypoxia-induced apoptosis in this lung cancer cell line. Although HIF-1α was found to be protective against hypoxia-induced apoptosis in cardiomyocytes, we instead found that increased HIF-1α expression further exacerbated apoptosis in A549 lung adenocarcinoma cells. We also found that inhibition of glycolysis with 2-DG attenuated HIF-1α-induced apoptosis in these cells during hypoxia. Both increased glycolysis and the resulting apoptosis could be inhibited by knocking-down HIF-1α expression with siRNA. Together, our results strongly implicate HIF-1α in the hypoxia-induced apoptosis of A549 cells, which depends on the glycolysis pathway and acidosis of the culture medium.

Despite examples to the contrary, this effect is not entirely unprecedented. In another relevant study, Schmaltz et al [10] reported that apoptosis in hypoxic cultures was primarily due to decreased pH of the culture medium, most likely caused by lactic acidosis. Here, we also observed that decreased pH triggered apoptosis in A549 cells. We were able to partially inhibit HIF-1α-induced apoptosis by increasing the buffering capacity of the medium to reduce acidosis. Further pointing to a role for HIF-1α in these processes, siRNA-knockdown of HIF-1α significantly blocked by the decrease of pH and apoptosis induced by hypoxia.

Consistent with our results, Krick et al [18] recently reported that hypoxia suppressed alveolar epithelial cell proliferation and enhanced alveolar type II cell apoptosis through activation of the HIF-1α/HRE axis and BNIP3, but they did not further investigate the role of acidosis in the action of BNIP3. Kubasiak et al [19], however, demonstrated that BNIP3 protein accumulated more rapidly under acidic pH and peaked at a significantly higher level than at neutral pH when cells were exposed to hypoxia. Acidosis was found to increase binding of BNIP3 to mitochondrial membranes, leading to apoptosis of hypoxic cells.

Taken together, when the cells are exposed to hypoxia, up-regulated expression of HIF-1α accelerates the glycolysis, which leads to the intracellular acidosis. Intracellular acidosis may activate the BNIP3 and promoted the hypoxic cell apoptosis. But this mechanism of HIF-1α on the hypoxia induced cell apoptosis needs further studies.

## Conclusion

Our findings indicate that HIF-1α plays a role in hypoxia-induced A549 apoptosis, and that this influence is dependent upon up-regulation of the glycolysis pathway and the resultant drop in pH of the culture medium.

## Competing interests

The author(s) declare that they have no competing interests.

## Authors' contributions

FML conceived of the experiments, carried out all experiments and prepared the manuscript. XJL conceived of the experiments and performed plasmid construction, RNA extraction, and real-time RT-PCR. NHY conceived of the experiments and constructed plasmid vectors. SQL performed cell culture and western-blotting. GQC performed RNA extraction and real-time RT-PCR. QYC performed cell culture and cell transfection. QJX provided expert advice and interpretation of the study's results. HJW performed TUNEL and flow-cytometry. All authors read and approved the final manuscript.

## Pre-publication history

The pre-publication history for this paper can be accessed here:
